# The Prevalence and Polymorphisms of Zonula Occluden Toxin Gene in Multiple *Campylobacter* *concisus* Strains Isolated from Saliva of Patients with Inflammatory Bowel Disease and Controls

**DOI:** 10.1371/journal.pone.0075525

**Published:** 2013-09-23

**Authors:** Vikneswari Mahendran, Ye Sing Tan, Stephen M. Riordan, Michael C. Grimm, Andrew S. Day, Daniel A. Lemberg, Sophie Octavia, Ruiting Lan, Li Zhang

**Affiliations:** 1 The School of Biotechnology and Biomolecular Sciences, University of New South Wales, Sydney, Australia; 2 Gastrointestinal and Liver Unit, the Prince of Wales Hospital, Sydney, Australia; 3 St George Clinical School, University of New South Wales, Sydney, Australia; 4 Department of Gastroenterology, Sydney Children’s Hospital, Sydney, Australia; 5 Department of Paediatrics, University of Otago, Christchurch, New Zealand; 6 School of Women’s and Children’s Health, University of New South Wales, Sydney, Australia; 7 Faculty of Medicine, University of New South Wales, Sydney, Australia; University of Aberdeen, United Kingdom

## Abstract

*Campylobacter* *concisus* is an oral bacterium. A number of studies detected a significantly higher prevalence of *C* *. concisus* in the intestinal tract of patients with inflammatory bowel disease (IBD) as compared to controls. The prevalence of zonula occluden toxin (zot) gene, which encodes a toxin known to increase intestinal permeability, in oral *C* *. concisus* strains is unknown. Increased intestinal permeability is a feature of IBD. A total of 56 oral *C* *. concisus* strains isolated from 19 patients with IBD and 20 controls were examined (some individuals were colonized with multiple strains). A filtration method was used for isolation of *C* *. concisus* from saliva samples. SDS-PAGE was used to define strains. PCR was used to amplify *zot* from *C* *. concisus* strains. Positive PCR products were sequenced and the nucleotides and amino acids were compared. Of the 56 oral *C* *. concisus* strains examined, 17 strains (30.4%) were positive for *zot*. The prevalence of *zot*-positive oral *C* *. concisus* strains was 54.5% in patients with active IBD, which was not significantly different from that in healthy controls (40%). Polymorphisms of *C* *. concisus* *zot* were revealed. *zot* ^808T^ *, zot* ^350-351AC^ and *zot* ^Multiple^ were detected only in patients with IBD, but not in healthy controls. Both *zot* ^808T^ and *zot* ^Multiple^ alleles resulted in substitution of valine at position 270, which occurred in 36.4% of patients with active IBD but not in healthy controls (*P* = 0.011). Furthermore, the prevalence of multiple oral *C* *. concisus* strains in patients with active IBD was significantly higher than that in healthy controls (*P* = 0.013). This is the first study reporting the prevalence of *zot* in human oral *C* *. concisus* strains and the polymorphisms of *C* *. concisus* *zot* gene. The data suggest that the possible role of *C* *. concisus* strains containing specific polymorphic forms of *zot* gene in human IBD should be investigated.

## Introduction

Increased *Campylobacter* *concisus* intestinal colonization has been associated with inflammatory bowel disease (IBD). IBD is a chronic inflammatory disease of the gastrointestinal tract with unknown aetiology. Crohn’s disease (CD) and ulcerative colitis (UC) are the two major clinical forms of IBD [1]. A number of studies have detected a significantly higher prevalence of *C* *. concisus* in fecal samples and intestinal biopsies collected from patients with IBD as compared to controls [2,3,4,5]. However, a recent study by Hansen et al. found a similar prevalence of *C* *. concisus* in intestinal biopsies collected from children with IBD and controls [6]. The difference in biopsy collection site (inflamed area vs macroscopically non-inflamed area at the edge of the inflamed site) may have contributed to the inconsistent results between the study from Hansen et al. and the other study in pediatric population [2].

*C* *. concisus* is a flagellated Gram-negative bacterium that requires H_2_-enriched microaerobic conditions for growth [7]. Humans are the main natural host of *C* *. concisus* , with the oral cavity being the primary colonization site [8,9]. We previously isolated *C* *. concisus* from 75% (44/59) of saliva samples from healthy individuals using a filtration method and detected *C* *. concisus* in 97% (57/59) of these samples using PCR [8]. In addition to the human oral cavity, *C* *. concisus* was detected by PCR in 12.5% of saliva sample of domestic cats [10]. *C* *. concisus* was also isolated from 10% (18/185) of chicken meat and 3% of beef meat (6/186) samples [11].

Using multilocus analysis of housekeeping genes, we showed *C* *. concisus* colonizing the human oral cavity to be a source of *C* *. concisus* that colonizes the human intestinal tract in some patients with IBD [12]. We also found that some patients with IBD are colonized with multiple *C* *. concisus* strains in the oral cavity and intestinal tract [12].

The mechanisms by which *C* *. concisus* may contribute to enteric diseases have been investigated. Both oral and enteric *C* *. concisus* strains have been shown to induce the production of IL-8 in HT-29 cells [13,14,15]. Some oral *C* *. concisus* strains isolated from patients with IBD were found to be invasive to Caco_2_ cells and more effective in upregulating surface expression of Toll like receptor 4 in HT-29 cells [12,15]. Furthermore, increased intestinal epithelial apoptosis and permeability by some *C* *. concisus* strains have been previously reported [13,14,16]. These data suggest that some oral *C* *. concisus* strains may have the potential to cause enteric diseases in individuals whose intestinal environment is suitable for *C* *. concisus* colonization.

Zonula occluden toxin (zot) gene has been detected in some *C* *. concisus* strains isolated from diarrheal and non-diarrheal stool samples [14]. The *zot* gene was first detected in *Vibrio cholerae*, the pathogen that causes cholera [17]. The *zot* gene in *V. cholerae* is part of a chromosomally integrated filamentous phage genome [18]. The protein encoded by

*V. cholerae zot* gene has been shown to increase intestinal permeability by affecting the tight junctions through actin reorganization [19]. Furthermore *V. cholerae zot* gene is related to induction of mild to moderate diarrhea [20]. Evidence suggests that increased intestinal permeability is a possible etiologic factor of IBD [21,22,23,24].

Currently, the prevalence of *zot* gene in human oral *C* *. concisus* strains is unknown. In this study, we have examined the prevalence of the *zot* gene in multiple *C* *. concisus* strains isolated from saliva samples of patients with IBD and controls. Furthermore, the polymorphisms of *C* *. concisus* *zot* gene were examined.

## Materials and Methods

### Ethics statement

Written informed consent was obtained from the adult subjects and the guardians on behalf of the minors/children involved in this study. Ethics approval for this study was granted by the Ethics Committees of the University of New South Wales and the South East Sydney Local Health District, Australia (HREC 09237/SESIAHS 09/078, HREC08335/SESIAHS (CHN) 07/48) and HREC 06233/SESAHS (ES) 06/164).

### Clinical information of patients with IBD and controls

Nineteen patients with IBD (13 CD and six UC) and 20 healthy controls recruited from Sydney area in Australia were included in this study. The patients were aged 5-73 years old and the controls were 4-67 years old. The age of patients with IBD (mean ± SD, 33 ± 5.7) and the healthy controls (26 ± 4.7) was not statistically different. Eleven patients had active disease (ten new cases and one relapsed case) and eight patients were in remission (Table 1). Patients with active disease were not receiving any treatment for IBD at the time of saliva sample collection and had not received antibiotics in the three month prior to sample collection. The relapsed patient with active disease (patient No. 2) had antibiotic treatment (metronidazole + ciprofloxacin) two years earlier, but not at the time of relapse. Five of the eight patients in remission had previously received antibiotics and all patients in remission were receiving immunosuppressive therapies at the time of sample collection. The details of the drugs received by patients in remission were listed in Table 2. The healthy controls had not received antibiotics in the three month prior to sample collection.

**Table 1:** Clinical information of patients with IBD included in this study.

**Patient ID**	***Age(y***)***/sex***	**Diagnosis**	**Disease activity**
**Patient No. 1**	5/M	CD	Remission
**Patient No. 2**	19/M	CD	Relapse, active
**Patient No. 3**	23/M	UC	New case, active
**Patient No. 4**	16/F	CD	Remission
**Patient No. 5**	13/M	CD	Remission
**Patient No. 6**	13/M	CD	Remission
**Patient No. 7**	65/M	UC	New case, active
**Patient No. 8**	16/M	CD	Remission
**Patient No. 9**	17/M	CD	Remission
**Patient No. 10**	19/M	CD	New case, active
**Patient No. 11**	33/M	CD	New case, active
**Patient No. 12**	55/F	CD	New case, active
**Patient No. 13**	22/M	UC	New case, active
**Patient No. 14**	34/M	UC	New case, active
**Patient No. 15**	39/M	UC	New case, active
**Patient No. 16**	67/M	UC	New case, active
**Patient No. 17**	73/M	CD	New case, active
**Patient No. 18**	14/F	CD	Remission
**Patient No. 19**	9/M	CD	Remission

Disease activity refers to the disease activity at the time of saliva sample collection.

**Table 2:** Treatment details of patients in remission.

**Patient ID**	**Diagnosis**	**Previous antibiotics**	**Current Treatment**
**Patient No. 1**	CD	No	Mesalazine
			Azathioprine
			Iron supplements
**Patient No. 4**	CD	No	Azathioprine
**Patient No. 5**	CD	Metronidazole	Mesalazine
		Ciprofloxacin	Azathioprine
		10 months prior	
**Patient No. 6**	CD	No	Mesalazine
**Patient No. 8**	CD	Metronidazole	Sulfasalazine
		2 months prior	
**Patient No. 9**	CD	Cotrimoxazole	Cotrimoxazole
		3 months prior	Tacrolimus
			Calcium
			Fish oil
**Patient No. 18**	CD	Metronidazole	Azathioprine
		Ciprofloxacin	
		1 year prior	
**Patient No. 19**	CD	Metronidazole	Mesalazine
		3 months prior	Azathioprine
			Iron supplements

Current treatment is the treatment that the patients were receiving at the time of sample collection.

### Isolation of multiple *C* *. concisus* strains from saliva samples

Isolation of *C* *. concisus* from saliva samples was carried out using a previously described filtration method [8]. For each saliva sample, 12 putative *C* *. concisus* isolates were collected. The putative *C* *. concisus* isolates were subjected to a previously described *C* *. concisus* PCR to confirm the identity of *C* *. concisus* and then subjected to sodium dodecyl sulphate polyacrylamide gel electrophoresis (SDS-PAGE) for whole cell protein profile analysis to define the strains as previously described [12]. Isolates with identical SDS-PAGE pattern were defined as the same strain.

### Detection of *zot* gene in *C* *. concisus* strains and sequencing the amplified *zot* gene

*C* *. concisus* DNA was prepared using the Puregene DNA Extraction kit (Gentra, Minneapolis, USA) following the manufacturer’s instructions. The forward primer FCCC13826_2075 (5’-TGCAAACCCTTTGTGATGAA-3’) has been previously described [14]. The reverse primer Ccon_*zot*R_257 (5’-TCGGTCCTCCACGATCTG-3’) was designed in this study using Primer 3 plus software (http://www.bioinformatics.nl/cgi-bin/primer3plus/primer3plus.cgi/) based on the genome sequence of *C* *. concisus* strain 13826 (Accession No. CP000792.1). PCR product size is 1055 base pairs (bp).

To amplify the *zot* gene, hot start PCR reactions were performed in a 25 µl reaction mixture containing PCR buffer, 200 nM of deoxynucleotide triphosphate, 2.5 mM MgCl_2_, 5.5 U of Taq polymerase (Fisher Biotech, Subiaco, Australia), 10 pmol of each primer and 10 ng of bacterial DNA extracted from each *C* *. concisus* strains. The thermal cycling conditions consist of denaturing at 96°C for 5 minutes, followed by 35 cycles of 95 °C for 10 seconds, annealing at 55–58 °C for 10 seconds and 72 °C for 45 seconds. All positive PCR products were sequenced from both ends using BigDye^TM^ reagents version 3.2 (Applied Biosystems, Foster City, CA) and analyzed on an ABI Capillary DNA Sequencer ABI3730 (Applied Biosystems). DNA extracted from *C* *. concisus* strain 13826 as the positive control and PCR mixture without bacterial DNA was used as the negative control.

### Analysis of *zot* gene sequences

Molecular evolutionary genetics analysis (MEGA) software version 5.0 was used for *zot* gene sequence alignment [25]. PHYLogeny Inference Packge (PHYLIP) was used to generate the neighbour-joining dendrogram of the *zot* gene amplified from different *C* *. concisus* strains [26]. Translation of nucleotide sequences was performed using Expasy translate tool [27]. Alignment of amino acid sequences was conducted using Clustalw multiple alignment tool [28].

### GenBank sequence submission

The sequences of the *zot* gene amplified from *C* *. concisus* strains were submitted to Genbank.

### Statistical analysis

Fisher’s exact test (two tailed) was used to compare the prevalence of multiple *C* *. concisus* strains in patients with IBD and controls and the prevalence of *zot* gene in *C* *. concisus* strains isolated from patients with IBD and control. Unpaired t test was used to compare the age of patients and controls. Statistical analysis was performed using GraphPad Prism 6 software (San Diego, CA).

## Results

### Isolation of multiple *C* *. concisus* strains from saliva samples of patients with IBD and controls

Of the 420 putative *C* *. concisus* isolates collected from saliva samples of 16 patients with IBD and 19 controls, 401 isolates were confirmed to be *C* *. concisus* by the *C* *. concisus* specific PCR. These 401 *C* *. concisus* isolates were shown to represent 50 different strains, with each strain showing a distinct whole cell protein profile on SDS-PAGE. These 50 oral *C* *. concisus* strains and the six oral *C* *. concisus* strains previously isolated from three patients with IBD and a healthy control were included in this study [12].

More than one oral *C* *. concisus* strains were isolated from each of nine patients with IBD and three controls (Table 3). The prevalence of multiple oral *C* *. concisus* strains in patients with active IBD was 63.6% (7/11), which was significantly higher than that in healthy controls 15% (3/20) in healthy controls (*P* = 0.013). Of the eight patients in remission, patients without antibiotics treatment had a prevalence of multiple oral *C* *. concisus* strains of 66.7% (2/3), none of the five patients who had antibiotics treatment were colonized with multiple oral *C* *. concicus* strains (0/5) (Tables 2 and 3).

**Table 3:** Individuals who were colonized with multiple oral *C* *. concisus* strains.

**Individual**	** *C* *. concisus* strains**
**Patient No. 1^#^**	P1CDO2, P1CDO3
**Patient No. 2^@^**	P2CDO3, P2CDO4
**Patient No. 4^#^**	P4CDO-S1, P4CDO-S2, P4CDO-S3
**Patient No. 10^@^**	P10CDO-S1, P10CDO-S2
**Patient No. 12^@^**	P12CDO-S1, P12CDO-S2
**Patient No. 13^@^**	P13UCO-S1, P13UCO-S2, P13UCO-S3
**Patient No. 14^@^**	P14UCO-S1, P14UCO-S2, P14UCO-S3
**Patient No. 15^@^**	P15UCO-S1, P15UC-SO2, P15UC-SO3
**Patient No. 16^@^**	P16UCO-S1, P16UCO-S2
**Healthy No. 8**	H8O-S1, H8O-S2
**Healthy No. 9**	H9O-S1, H9O-S2, H9O-S3
**Healthy No. 11**	H11O-S1, H11O-S2

^@^ IBD patients with active disease. ^#^ IBD patients in remission. Nine patients with IBD and three healthy controls were colonized with multiple oral *C* *. concisus* strains. The remaining 10 patients with IBD and 17 healthy controls were colonized with a single *C* *. concisus* strain in the oral cavity. The strains were defined by *C* *. concisus* specific PCR and SDS-PAGE patterns. The prevalence of multiple oral *C* *. concisus* strains was 63.6% (7/11) in patients with active IBD, which was significantly higher than that in healthy controls 15% (3/20) (*P* = 0.013). In patients in remission, the prevalence of multiple oral *C* *. concisus* strains was 66.7% (2/3) in patients without antibiotics treatment and was zero (0/5) in patients received antibiotics treatment.

### The prevalence of *zot* gene in oral *C* *. concisus* strains isolated from patients with IBD and controls

Of the total 56 oral *C* *. concisus* strains examined in this study, 17 strains (30.4%) were positive for *zot* gene. In individuals colonized with multiple oral *C* *. concisus* strains, usually only one strain was positive for *zot* gene except for patient No. 2 and patient No. 13. The two oral *C* *. concisus* strains isolated from patient No. 2 were positive for *zot* gene and two of the three strains isolated from patient No. 13 were positive for *zot* gene.

**Figure 1:**
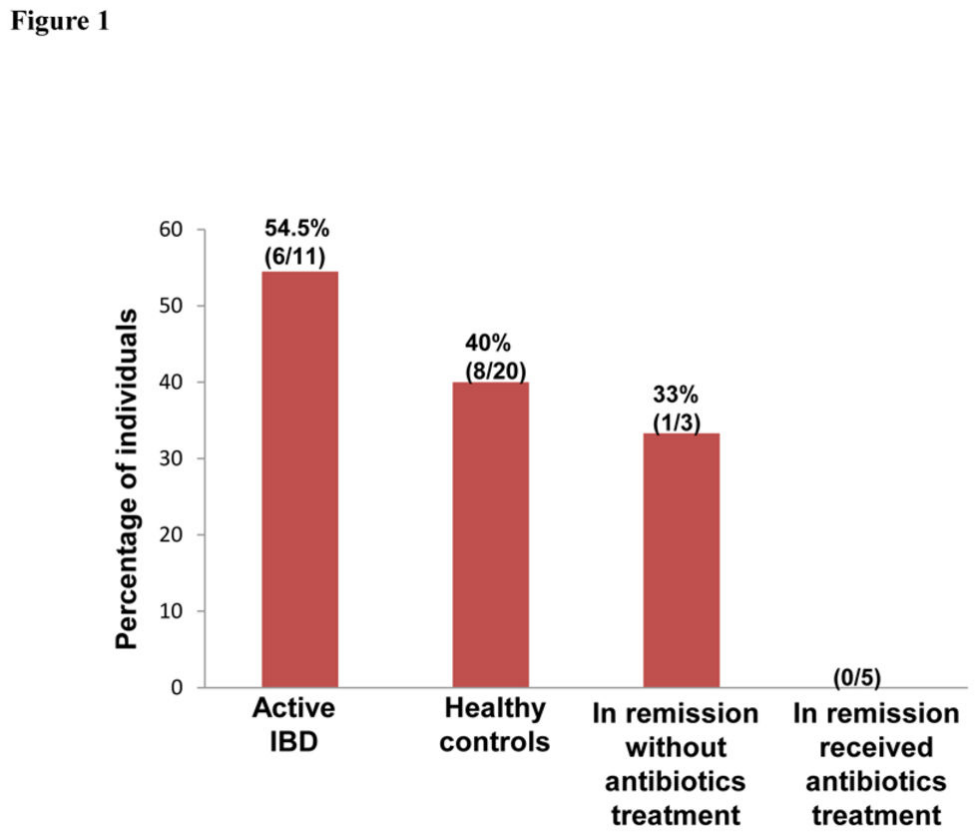
Prevalence of *zot*-positive *C* *. concisus* strains in the oral cavity of patients with IBD and controls. The prevalence of *zot*-positive *C* *. concisus* strains in active IBD and healthy controls was not statistically different. *zot*-positive *C* *. concisus* strains were not detected in patients in remission who received antibiotics treatment for IBD.

The prevalence of *zot*-positive *C* *. concisus* strains in the oral cavity of healthy controls was 40% (8/20). The prevalence of *zot*-positive *C* *. concisus* strains in healthy children was 50% (4/8), which was not statistically different from that in the adult healthy individuals (33%, 4/12). The prevalence of *zot*-positive *C* *. concisus* strains in the oral cavity of patients with active IBD was 54.5% (6/11), which was not significantly higher than that in healthy controls (40%, 8/20). Of the eight patients in remission, patients without antibiotics treatment for IBD had a prevalence of *zot*-positive oral *C* *. concisus* strains of 33.3% (1/3), none of the five patients who had antibiotics treatment were colonized with *zot*-positive oral *C* *. concicus* strains (0/5) (Figure 1).

### Comparison of the sequences of *zot* gene amplified from oral *C* *. concisus* strains isolated from patients with IBD and controls

**Figure 2:**
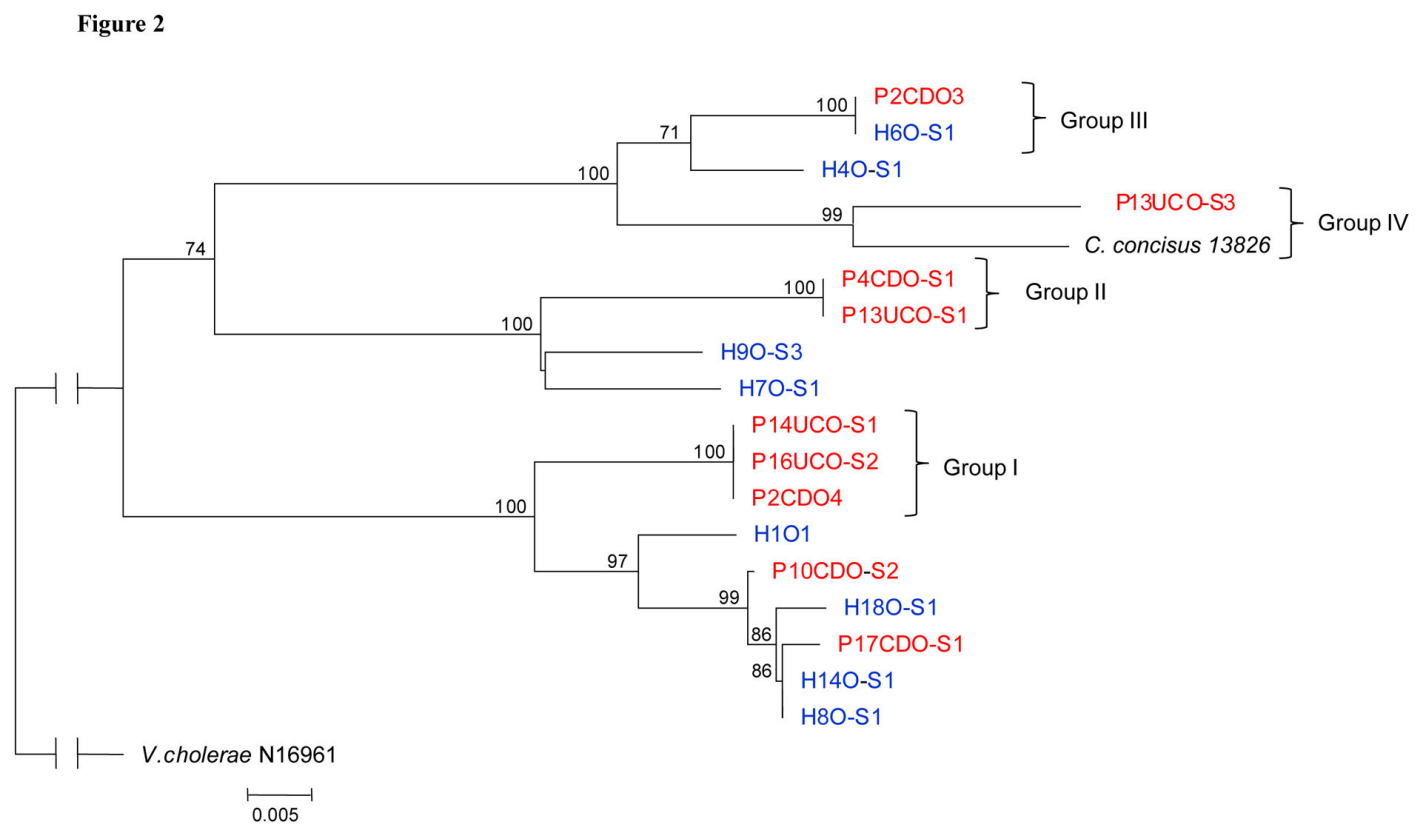
Neighbour-joining dendrogram based on the *zot* sequences of 17 oral *C* *. concisus* strains. Strains from patients with IBD are coloured red. Strains from healthy controls are coloured blue. Groups I, II and III are identical. *Vibrio cholerae* is used as an outgroup. *C* *. concisus* strain 13826 is the whole genome sequenced strain (Accession No. CP000792.1). P2CDO3 and P2CDO4 were from patient No. 2. P13UCO-S1 and P13UCO-S3 were from patient No. 13. The remaining strains were from individual patients and controls.

The neighbour- joining dendrogram generated based on the DNA sequences of the sequences of *zot* gene amplified from the 17 *zot-*positive *C* *. concisus* strains (670 bp) is shown in Figure 2. The whole genome sequenced *C* *. concisus* 13826 was also included in the analysis. Three groups of *C* *. concisus* *zot* with identical *zot* gene sequences (Group I, Group II and Group III) were revealed. *C* *. concisus* strains in Group I and Group II were from patients with IBD only. In Group III, one strain was from a patient with IBD and one strain was from a healthy control.

In addition to the three groups of *C* *. concisus* with identical *zot* gene sequences, another group of *zot* (Group IV), which contained *C* *. concisus* strains from individuals with enteric diseases, was identified. Group IV consisted of one oral strain from a patient with UC and one enteric strain from a patient with bloody diarrhea ( *C* *. concisus* strain 13826), the similarity of these two *zot* gene sequences was 95%.

### Genetic polymorphisms of the *zot* gene in different oral *C* *. concisus* strains

The nucleotide sequences of the *zot* gene from the 17 *zot-*positive *C* *. concisus* strains were aligned to examine the nucleotide polymorphisms (Table 4). Group I *C* *. concisus* *zot* had a unique nucleotide polymorphism at position 808bp (*zot* ^808T^) and Group II *C* *. concisus* *zot* had unique nucleotide polymorphisms at positions 350bp and 351bp (*zot* ^350-351AC^) ( *C* *. concisus* strain 13826 nucleotide position). Group III *C* *. concisus* *zot* did not show any unique polymorphisms. Group IV *C* *. concisus* *zot* had nucleotide polymorphisms at ten positions including 747 bp, 769 bp, 786-789 bp, 805-806 bp, 809 bp and 816 bp (*zot* ^Multiple^). The *zot* genes amplified from oral *C* *. concisus* strains isolated from healthy controls did not show any unique nucleotide polymorphisms (Table 4).

**Table 4:** Nucleotide polymorphisms of Group I, Group II and Group IV *C* *. concisus* *zot*.

	**350-351^*a*^**	**747**	**769**	**786-789**	**805-806**	**808-809**	**816**
** *C* *. concisus* strains**							
**Group I**	CA	T	A	GCCT	AG	**T**T	T
**Group II**	**AC**	T	A	GCCT	AG	GT	T
**Group III**	CG	T	A	GCCT	AG	GT	T
**Group IV**	CG	**A**	**G**	**TATA**	**GA**	G**C**	**A**
**H9O-S3**	CG	T	A	GCCT	AG	GT	T
**H7O-S1**	CG	T	A	GCCT	AG	GT	T
**P17CDO-S1**	CA	T	A	ACCT	AG	GT	T
**H1O1**	CA	T	A	ACCT	AG	GT	T
**H8O-S1**	CA	T	A	ACCT	AG	GT	T
**H14O-S1**	CA	T	A	ACCT	AG	GT	T
**P10CDO-S1**	CA	T	A	GCCT	AG	GT	T
**H18O-S1**	CA	T	A	GCCT	AG	GT	T
**H4O-S1**	CG	T	A	GCCT	AG	GT	T

Genetic polymorphisms are in bold and underlined. ^a^ The numbers indicate the nucleotide positions of *C* *. concisus* *zot* gene. Group I *C* *. concisus* *zot* had a unique nucleotide polymorphism at position 808 bp (*zot* ^808T^). Group II *C* *. concisus* *zot* had unique nucleotide polymorphisms at positions 350 bp and 351 bp (*zot* ^350-351AC^). Group IV *C* *. concisus* *zot* had nucleotide polymorphisms at ten positions including 747 bp, 769 bp, 786-789 bp, 805-806 bp, 809 bp and 816 bp (*zot* ^Multiple^). The *zot* genes amplified from the remaining

*C* *. concisus* strains did not show any unique nucleotide polymorphisms.

### Amino acid polymorphisms of different alleles of *C* *. concisus* *zot* gene

The amino acids encoded by the *zot* gene of the 17 *zot-*positive *C* *. concisus* strains were aligned to examine whether the nucleotide polymorphisms of the above three *zot* alleles (*zot* ^808T^, *zot* ^350-351AC^ and *zot* ^Multiple^) have resulted in changes of amino acids. *zot* ^808T^ resulted in the change of valine to leucine at position 270 ( *C* *. concisus* strain 13826 amino acid position). *zot* ^350-351AC^ resulted in the change of threonine to asparagine at position 117. *zot* ^Multiple^ allele resulted in unique amino acids at seven positions, including aspartic acid to glutamic acid at position 249, threonine to alanine at position 257, having an asparagine at position 262 which was different from all other *C* *. concisus* strains, proline to isoleucine at position 263, serine to aspartic acid at position 269, valine to alanine at position 270 and asparagine to lysine at position 272. Both *zot* ^808T^and *zot* ^Multiple^ resulted in substitution of valine at position 270 (Table 5).

**Table 5:** Amino acid polymorphisms encoded by Group I, Group II and Group IV *C* *. concisus* *zot*.

	**117** ^*a*^	**249**	**257**	**262**	**263**	**269**	**270**	**272**
** *C* *. concisus* strains**								
**Group I**	T	D	T	K	P	S	**L**	N
**Group II**	**N**	D	T	E	P	S	V	N
**Group III**	T	D	T	E	P	S	V	N
**Group IV**	T	**E**	**A**	**N**	**I**	**D**	**A**	**K**
**H9O-S3**	T	D	T	E	P	S	V	N
**H7O-S1**	T	D	T	E	P	S	V	N
**P17CDO-S1**	T	D	T	T	P	S	V	N
**H1O1**	T	D	T	T	P	S	V	N
**H8O-S1**	T	D	T	T	P	S	V	N
**H14O-S1**	T	D	T	T	P	S	V	N
**P10CDO-S1**	T	D	T	K	P	S	V	N
**H18O-S1**	T	D	T	K	P	S	V	N
**H4O-S1**	T	D	T	E	P	S	V	N

Amino acid polymorphisms are in bold and underlined. ^a^ The numbers indicate the amino acid positions of *C* *. concisus* Zot protein. *zot* ^808T^ resulted in the change of valine to leucine at position 270. *zot* ^350-351AC^ resulted in the change of threonine to asparagine at position 117. *zot* ^Multiple^ allele resulted in unique amino acids at seven positions.

### The association between *C* *. concisus* *zot* polymorphisms and IBD

*zot* ^808T^ *, zot* ^350-351AC^ and *zot* ^Multiple^ alleles were detected only in patients with IBD, not in healthy controls (Table 6). The prevalence of *zot* ^808T^ allele in patients with active IBD (27.2%, 3/11) was significantly different from that in healthy controls (0/20) (*P* = 0.037) (Table 6). *zot* ^350-351AC^ allele was detected in 9% of patient with active IBD (1/11) and none of the healthy controls. *zot* ^Multiple^ was detected in 9% of patient with active IBD (1/11) and none of the healthy controls.

**Table 6:** Prevalence of *zot* ^808T^, *zot* ^350-351AC^ and *zot* ^Multiple^ in patients with IBD and controls.

	**Active IBD n=11**	**Healthy controls n=20**	**IBD in remission without antibiotics treatment n=3**	**IBD in remission received antibiotics treatment n=5**
***zot*^808T^**	3/11 (27.2%)*^@^	0/20	0/3	0/5
***zot*^350-351AC^**	1/11 (9%)	0/20	1/3 (33%)	0/5
***zot*^Multiple^**	1/11 (9%)^@^	0/20	0/3	0/5

*zot* ^808T^ *, zot* ^350-351AC^ and *zot* ^Multiple^ were detected only in patients with IBD, not in healthy controls. * The prevalence of *zot* ^808T^ allele in patients with active IBD (27.2%) was significantly higher compared to healthy controls (0/20) (*P* = 0.037). ^@^ Polymorphisms of *zot* that have resulted in substitution of valine at position 270, which was detected only in patients with active IBD (36.4%, 4/11) but not in healthy controls (0/20) (*P* = 0.011).

Of the eight patients in remission, one of the three patients who received no antibiotics treatment for IBD was colonized with a *zot* ^350-351AC^ strain. *zot* ^808T^ *, zot* ^350-351AC^ and *zot* ^Multiple^ alleles were not detected in any of the five patients who received antibiotics treatment for IBD (Table 6).

At the amino acid level, substitution of valine at position 270 occurred in 36.4% of patients with active IBD (4/11) and none of the healthy controls (0/20) (*P* = 0.011). Substitution of valine at position 270 was not detected in *C* *. concisus* strains isolated from patients in remission (Tables 5 and 6).

### Sequence accession numbers

The accession numbers for the sequences of *zot* gene submitted to GenBank were KC935342-KC935358.

## Discussion

In this study, we investigated the presence of *zot* gene in multiple *C* *. concisus* strains isolated from saliva samples of patients with IBD and healthy controls and the polymorphisms of *C* *. concisus* *zot* gene.

The toxin encoded by *V. cholerae zot* gene affects the tight junctions through activation of proteinase activated receptor 2, which results in an increased intestinal epithelial permeability [29]. Patients with IBD have an increased intestinal permeability in comparison to healthy controls [21,22,23,24]. The increase in intestinal permeability has been found to precede the development of inflammatory changes, suggesting that increased intestinal permeability is a possible etiologic factor of IBD [21,22,23]. Zeissig et al. found that there was a change in expression and distribution of tight junction proteins in patients with active CD as compared to controls [30]. In this study we detected three *C* *. concisus* *zot* alleles (*zot* ^808T^, *zot* ^350-351AC^ and *zot* ^Multiple^ alleles) only in patients with IBD, but not in healthy controls. Interestingly, both *zot* ^808T^ and *zot* ^Multiple^ alleles resulted in substitution of valine at position 270 and substitution of valine at position 270 was detected only in patients with active IBD. These data suggest that specific mutations have occurred in the *zot* gene of *C* *. concisus* strains isolated from patients with active IBD. It is possible that these mutations have changed the function of *C* *. concisus* Zot toxin or increased the interaction between the *C* *. concisus* Zot toxin with human intestinal epithelial cells, which contributes to the initiation of IBD. These speculations remain to be investigated in future studies.

In addition to its association with IBD, *C* *. concisus* has been frequently isolated from diarrheal stool samples [31,32,33,34,35]. Recently, Nielsen et al. found that gastroenteritis caused by *C* *. concisus* was milder than that caused by *Campylobacter jejuni* and *Campylobacter coli* [34]. The level of fecal calprotecin, a marker of inflammation, in individuals colonized with *C* *. concisus* was similar to the level seen in patients with viral gastroenteritis [35]. In addition to causing an increase in intestinal epithelial permeability, toxin encoded by *Vibrio cholerae zot* has also been associated with mild to moderate diarrhea in humans [20]. It is possible that some polymorphic forms of *C* *. concisus* *zot* may play a role in human diarrheal disease, which requires further investigation.

We performed bioinformatics analysis of the whole genome sequenced *C* *. concisus* strain 13826 and found that the *zot* gene is a component of a prophage genome (unpublished data). Our finding in this study that approximately 30% of *C* *. concisus* strains colonizing the human oral cavity were positive for *zot* suggests that these *C* *. concisus* strains were infected with bacterial phage. Of the multiple *C* *. concisus* strains isolated from individual patients with IBD and controls, usually only one *C* *. concisus* strain was positive for *zot* (except for patient No. 2 and patient No. 13). This suggests that *zot*-positive *C* *. concisus* strains may have specific receptors that predispose them to bacterial phage infection.

A further interesting finding from this study was that a significantly higher number of IBD patients with active disease were colonized with multiple oral *C* *. concisus* strains in comparison to healthy controls (63.6% vs 15%). It is possible that patients with active IBD have acquired more virulent *C* *. concisus* strains from other sources in addition to the existing *C* *. concisus* strains that colonize the oral cavity. An alternative explanation is that *C* *. concisus* strains colonizing the oral cavity of patients with IBD may have gone through genetic changes and resulted in new *C* *. concisus* strains.

In our study, we have included eight IBD patients who were in remission. Data from this group of patients suggest that antibiotics used in treatment of IBD have effects on oral

*C* *. concisus* colonization. This view is supported by the findings that none of the five patients who received antibiotics treatment for IBD were colonized with multiple oral *C* *. concisus* strains and that there were no *zot*-positive *C* *. concisus* strains isolated from these patients. However, whether the antibiotics used in these five patients with IBD have eradicated *zot-*positive *C* *. concisus* strains from the oral cavity or greatly inhibited the *zot-*positive*C. concisus* strains is unknown.

The prevalence of *zot*-positive *C* *. concisus* in the human intestinal tract has not been systematically investigated. A study from Kalischuk et al. detected *zot* in 80% (4/5) of *C* *. concisus* strains isolated from stool samples of healthy controls and in 22% (2/9) of *C* *. concisus* strains isolated from stool samples of patients with diarrhea [14]. However, this study did not isolate multiple *C* *. concisus* isolates from individual patients or controls and detected only five *C* *. concisus* strains from healthy controls. Given this, it is difficult to draw a convincing conclusion regarding the prevalence of *zot*-positive intestinal *C* *. concisus* strains based on this study. Future studies examining larger numbers of enteric samples are required to determine the prevalence of *zot*-positive *C* *. concisus* strains in the intestinal tract of healthy individuals and patients with enteric diseases including IBD as well as the polymorphisms of *zot* gene in enteric *C* *. concisus* strains.

In addition to IBD, increased gut permeability has been seen in a number of other chronic human diseases such as diabetes [36]. Our findings that about 30% of oral *C* *. concisus* strains in the human oral cavity were positive for *zot* and *C* *. concisus* *zot* has polymorphic forms suggest that future studies should be conducted to investigate whether *C* *. concisus* *zot* contributes to the initiation of these chronic human diseases.

In summary, this is the first study examining the prevalence of *zot*-positive *C* *. concisus* strains in the human oral cavity and the polymorphisms of *C* *. concisus* *zot* gene. We found that about 30% of oral *C* *. concisus* strains in the human oral cavity were positive for *zot* and that *C* *. concisus* *zot* gene has polymorphic forms. *zot* ^808T^, *zot* ^350-351AC^ and *zot* ^Multiple^ alleles were detected only in patients with IBD, but not in the healthy controls. Both *zot* ^808T^ and *zot* ^Multiple^ alleles resulted in substitution of valine at position 270, which occurred only in patients with active IBD. Furthermore, a significantly higher number of patients with active IBD were colonized with multiple oral *C* *. concisus* strains as compared to healthy controls. These data suggest that future studies are required to investigate the effects of polymorphic *C* *. concisus* Zot proteins on human gastrointestinal tract permeability and their potential involvement in initiating a subgroup of human IBD. Despite the very interesting findings in this study, the sample size of patients with IBD was relatively small. Future studies examining a larger number of patients with IBD should be conducted to verify the findings of this study.
